# *De novo* intrachromosomal gene conversion from *OPN1MW* to *OPN1LW* in the male germline results in Blue Cone Monochromacy

**DOI:** 10.1038/srep28253

**Published:** 2016-06-24

**Authors:** Elena Buena-Atienza, Klaus Rüther, Britta Baumann, Richard Bergholz, David Birch, Elfride De Baere, Helene Dollfus, Marie T. Greally, Peter Gustavsson, Christian P. Hamel, John R. Heckenlively, Bart P. Leroy, Astrid S. Plomp, Jan Willem R. Pott, Katherine Rose, Thomas Rosenberg, Zornitza Stark, Joke B. G. M. Verheij, Richard Weleber, Ditta Zobor, Nicole Weisschuh, Susanne Kohl, Bernd Wissinger

**Affiliations:** 1 Institute for Ophthalmic Research, Centre for Ophthalmology, Tuebingen, Germany; 2 Sankt Gertrauden-Krankenhaus, Berlin, Germany; 3 Department of Ophthalmology, Charité – Universitätsmedizin Berlin, Campus Virchow-Klinikum, Berlin, Germany; 4 Retina Foundation of the Southwest, Tom and Dorothy Anderson Vision Research Center, Texas, USA; 5 Department of Ophthalmology & Center for Medical Genetics, Ghent University Hospital, Ghent, Belgium; 6 Centre de Référence pour les Affections Rares en Génétique Ophtalmologique, Hôpitaux Universitaires de Strasbourg, Strasbourg, France; 7 National Centre for Medical Genetics, Our Lady’s Children’s Hospital, Dublin, Ireland; 8 Department of Molecular Medicine and Surgery, Karolinska Institutet, Stockholm, Sweden; 9 Genetic Sensory Diseases - Hopital Gui de Chauliac, Centre Hospitalier Universitaire, Montpellier, France; 10 Department of Ophthalmology and Visual Sciences, W. K. Kellogg Eye Center, University of Michigan, Ann Arbor, MI, USA; 11 Department of Clinical Genetics, Academic Medical Center, Amsterdam, The Netherlands; 12 Department of Ophthalmology, University Medical Centre Groningen, University of Groningen, The Netherlands; 13 Genetic Health Services Victoria, Monash Medical Centre, Parkville, Australia; 14 National Eye Clinic for the Visually Impaired, Kennedy Center, Glostrup, Denmark; 15 Victorian Clinical Genetics Services, Murdoch Childrens Research Institute, Parkville, Victoria, Australia; 16 Department of Medical Genetics, University Medical Centre Groningen, University of Groningen, The Netherlands; 17 Department of Ophthalmic Genetics, Casey Eye Institute, Portland, OR, USA

## Abstract

X-linked cone dysfunction disorders such as Blue Cone Monochromacy and X-linked Cone Dystrophy are characterized by complete loss (of) or reduced L- and M- cone function due to defects in the *OPN1LW/OPN1MW* gene cluster. Here we investigated 24 affected males from 16 families with either a structurally intact gene cluster or at least one intact single (hybrid) gene but harbouring rare combinations of common SNPs in exon 3 in single or multiple *OPN1LW* and *OPN1MW* gene copies. We assessed twelve different *OPN1LW/MW* exon 3 haplotypes by semi-quantitative minigene splicing assay. Nine haplotypes resulted in aberrant splicing of ≥20% of transcripts including the known pathogenic haplotypes (i.e. ‘LIAVA’, ‘LVAVA’) with absent or minute amounts of correctly spliced transcripts, respectively. *De novo* formation of the ‘LIAVA’ haplotype derived from an ancestral less deleterious ‘LIAVS’ haplotype was observed in one family with strikingly different phenotypes among affected family members. We could establish intrachromosomal gene conversion in the male germline as underlying mechanism. Gene conversion in the *OPN1LW/OPN1MW* genes has been postulated, however, we are first to demonstrate a *de novo* gene conversion within the lineage of a pedigree.

The apo-proteins of the human long-wavelength and middle-wavelength sensitive cone photoreceptor pigments are encoded by the *OPN1LW* (*LW*; OMIM 300822) and *OPN1MW* genes (*MW*; OMIM 300821), respectively. Arranged in a head-to-tail tandem array on the long arm of the X-chromosome^1^, these duplicated genes share 98% sequence identity^2^. The prototypic gene array structure consists of a *LW* followed by a *MW* gene, however, considerable variability has been observed in gene copy number in the human *LW/MW* gene array^3^,^4^. Yet, studies on the human retina showed that only the two most proximal genes in the array are expressed at appreciable levels^5^. Duplicated genes are prone to unequal homologous recombination and gene conversion, the non-reciprocal transfer of genetic information in which a recipient sequence is replaced by a donor sequence that remains unaltered^6^. Concurrently, gene conversion and recombination themselves, enhance the interchange of DNA variants, thus reducing the divergence between duplicates while expediting a considerably high haplotype diversity^7^. In accordance with gene variability at the population level, both mechanisms are presumed to occur at the *LW/MW* gene cluster; nonetheless, they have never been directly observed.

Blue Cone Monochromacy (BCM, OMIM 303700) is an X-linked inherited cone dysfunction disorder. Affected subjects present with colour vision abnormalities and reduced visual acuity, but nystagmus and photophobia are also common^8^. In this condition S-cone function is retained, while the function of both L- and/or M-cones is absent. In the allelic condition, X-linked Cone Dystrophy (XLCD), cone function is strongly and progressively impaired. Such patients regularly present with myopia and astigmatism and dichromatic or monochromatic colour vision. The two most common genetic causes of BCM are large deletions encompassing the Locus Control Region (LCR) preventing the expression of the *LW/MW* genes^9^,^10^ or the presence of either a single *LW/MW* hybrid opsin gene or multiple *LW/MW* opsin genes inactivated by the p.C203R missense mutation^9^,^11^. Very few additional disease-causing point mutations or small internal deletions in the *LW/MW* array have been reported^12^,^13^,^14^,^15^.

A particular combination of common polymorphisms in *LW* exon 3 segregating in a single BCM family was first noted by Nathans and colleagues but not considered as disease-causing at that time^9^. This haplotype, referred to as ‘LIAVA’ in the literature, comprises the following SNPs: c.(453A > G; 457A > C; 465C > G; 511G > A; 513G > T; 521C; 532A > G; 538T > G) and a thereof deduced cone pigment variant with a certain combination of amino acid exchanges: p.[(=); M153L; (=); V171I; A174A; I178V; S180A]. Neither the spectral properties of this variant cone pigment nor its membrane trafficking is altered^16^. Still the ‘LIAVA’ haplotype has never been reported in individuals with normal colour vision but in patients with incomplete achromatopsia or XLCD^17^,^18^ and is associated with widespread alterations of the retinal morphology and disorganized cone structure, different from findings in BCM patients carrying the p.C203R mutation^19^. In a recent study involving subjects with protan colour vision defects, the ‘LIAVA’ haplotype and other rare exon 3 haplotypes in *LW* were found to induce exon 3 skipping *in vitro*^20^. These findings have lately been corroborated and extended to patients with severe cone disorders including BCM and the identification of two further haplotypes, ‘LVAVA’ and ‘MIAVA’, that impair splicing^21^.

Hitherto, the interplay of *cis*-regulatory elements and their contribution to exon 3 splicing is still unclear. To better ascertain to what extent different exon 3 haplotypes lead to splicing aberrations and thereby contribute to severe cone dysfunction disorders, we pursued a semi-quantitative assessment of transcripts from minigene assays performed on a comprehensive set of rare exon 3 haplotypes observed in a total of sixteen families diagnosed with BCM or XLCD. Gene conversion has been proposed as one mechanism underlying the formation of rare exon 3 haplotyopes. However, little is known about the specific features of such gene conversion in the human cone opsin genes. Taking advantage of a family with strikingly different ocular phenotype between the grandfather and grandson, we were able to identify an intrachromosomal *de novo* gene conversion event in the male germline which results in the replacement of a permissive haplotype by the strongly deleterious ‘LIAVA’ haplotype in the *LW* gene and explains the severe BCM phenotype in the grandson.

## Materials and Methods

### Patient recruitment and clinical evaluation

The study was performed in compliance with the tenets of the WMA Declaration of Helsinki. Study participants were recruited *ad hoc* at different centers specialized in inherited retinal diseases during routine clinical diagnostics. All participants gave written informed consent – approved by the respective local research and ethical review boards - for participation in the study for which blood or DNA samples were sent to Tuebingen for genetic analysis. Procedures of the genetic analysis were approved by the Ethics Committee of the Medical Faculty, Eberhard-Karls University Tuebingen. Patients underwent basic ophthalmologic examination according to the standards of the recruiting centers (for details see Supplementary information).

### Genotyping of the *LW/MW* gene cluster

Genomic DNA was isolated from blood samples according to standard procedures. We analyzed the basic structure and integrity of the *LW/MW* gene cluster and the absence of the common p.C203R mutation by means of an established PCR and PCR/RFLP protocol. For those subjects having a structurally intact array, *LW* or *MW* specific long distance PCRs (LD-PCRs) were performed and LD-PCR products sequenced. For subjects with multiple gene copies, the total number of *LW/MW* opsin genes was determined by means of real-time quantitative PCR (for details see Supplementary information).

### Minigene preparation

The prototype *LW* opsin minigene construct was kindly provided by Dr. Ueyama (Shiga University, Japan) and used to generate minigene constructs with *LW/MW* gene variants in exon 3, flanked by its native intronic sequences and the remaining human *LW* cDNA sequence (for details see Supplementary information).

### Transfection and RNA extraction

HEK293 cells at 80–90% confluency were transfected with 4 μg DNA of the minigene construct using 20 μl Lipofectamine 2000 (ThermoFischer GmbH, Dreieich, Germany) per well. 24 h post-transfection, total RNA was extracted (for details see Supplementary information).

### RT-PCR and relative quantification

First strand cDNA synthesis was performed using 2 μg of total RNA and random hexamer primers. Subsequent PCR was performed with a 5′ FAM (6-carboxyfluorescein) labeled forward primer and using the QIAGEN Multiplex PCR Kit (Qiagen, Hilden, Germany). FAM-labeled RT-PCR products were diluted 1:10 in water; mixed with 1 μl of GeneScan ROX500 size standard (Life Technologies, Darmstadt, Germany) and 8 μl of Hi-Di Formamide (Life Technologies) in a total volume of 10 μl. Mixes were separated by capillary electrophoresis on an ABI 3130XL Genetic Analyzer instrument (Life Technologies). The area-under-the-curve (AUC) was calculated with GeneMapper 5 (Life Technologies) software. Ratios of splicing products were determined as the AUC for individual peaks divided by the sum of AUC of all differentially spliced products.

### Microsatellite analysis

Microsatellite markers locating either centromeric (DXS8011, DXS8103, DXS1356, DXS8087) or telomeric (L441TA, L441CA, AF277A, AF277B and DXS1073) to the *LW/MW* cluster were genotyped in the three BCM72 family members. Markers for which the mother BCM72-II:1 was heterozygous were used to reconstruct haplotypes.

### Mapping of the gene conversion event

LD-PCRs were performed for all three members of BCM72 for the proximal gene copy and for the distal copies. Upon cloning of digested LD-PCR products, we selected two independent clones of the following gene copies for further analysis: (i) *LW*-derived clones bearing the ‘LIAVA’ haplotype from subjects BCM72-II:1 and BCM72-III:1, (ii) *MW*-derived clones bearing the ‘LIAVA’ haplotype from subject BCM72-I:1, and (iii) *LW*-derived clones bearing the ‘LIAVS’ haplotype from subject BCM72-I:1. We sequenced the clones by primer walking (for details see Supplementary information).

### Bioinformatic predictions and reference sequences

See Supplementary information.

## Results

### *LW/MW* genotypes of subjects with X-linked cone dysfunction disorders

**Figure 1:**
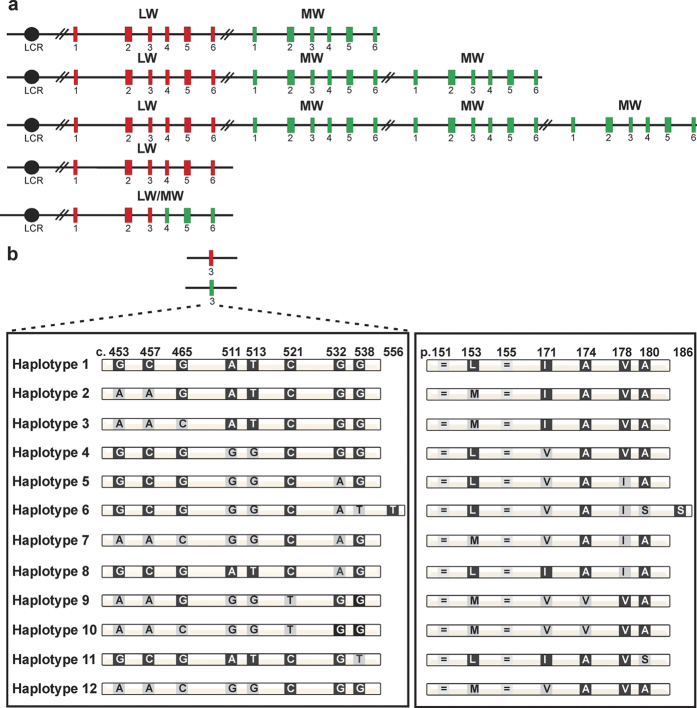
Structural diversity of the *LW/MW* gene array and exon 3 haplotypes in BCM and XLCD families analyzed in this study. (**a**) Overview of the various *LW/MW* gene arrays observed in the study cohort. The *LW/MW* gene cluster comprises the LCR, represented by a black circle, *LW* exons depicted as red and *MW* exons as green boxes, respectively. We observed single *LW* or *LW/MW* hybrid arrays and multiple gene arrays with one *LW* copy and one up to three *MW* copies. (**b**) The twelve different exon 3 haplotypes found in this cohort comprise eight common SNPs, depicted by the coding nucleotide position (left) and the corresponding variant amino acid residues (right) and a novel missense variant c.556C > T/p.P186S as part of haplotype 6.

We genotyped the *LW/MW* gene cluster in twenty-four affected subjects from sixteen families diagnosed with BCM or XLCD not carrying mutations commonly reported in BCM. Eleven families had structurally intact arrays with a proximal *LW* gene followed by one (n = 6) or multiple copies of the *MW* gene (n = 5). The remaining five families harboured either a single *LW* (n = 3) or a single *LW/MW* hybrid gene (n = 2) (Fig. 1a).

Sequencing of *LW/MW* exons revealed a high proportion of different (n=12) rare exon 3 haplotypes (Fig. 1b). Table 1 depicts the LW/MW gene array composition and the exon 3 haplotypes for each subject. For consistency we designate haplotypes according to the amino acid residues they encode (i.e. ‘LIAVA’ for p.[L153-I171-A174-V178-A180]). Exon 3 haplotypes comprise two synonymous variants, c.453A > G and c.465C > G. While the c.453A allele was in strict linkage disequilibrium with c.457A/p.V153, we observed both alleles of the variant c.465C > G in *cis* with c.457A/p.V152. For ease, we distinguish these alleles hereafter with a superscript add-on where appropriate (e.g. MIAVA^c.465C^ versus MIAVA^c.465G^). Except for a missense mutation c.556C > T/p.P186S found in the *LW* gene of subject BCM142–21958, all other variants comprised in the haplotypes are *per se* common in the population of colour normal subjects (minor allele frequency; MAF > 0.05; 22, 23). A large fraction of these haplotypes is rare: seven out of the twelve haplotypes observed here (Table 2) were not present in the population sample investigated by Winderickx and colleagues^22^. In all our sixteen families the opsin gene harbouring such a rare haplotype occupies the most proximal position with respect to the LCR. In subjects ZD379–19194, BCM101–19818, and BCM126–20616 also the distal gene copies bear rare exon 3 haplotypes.

### Minigene assay for rare exon 3 haplotypes

**Figure 2:**
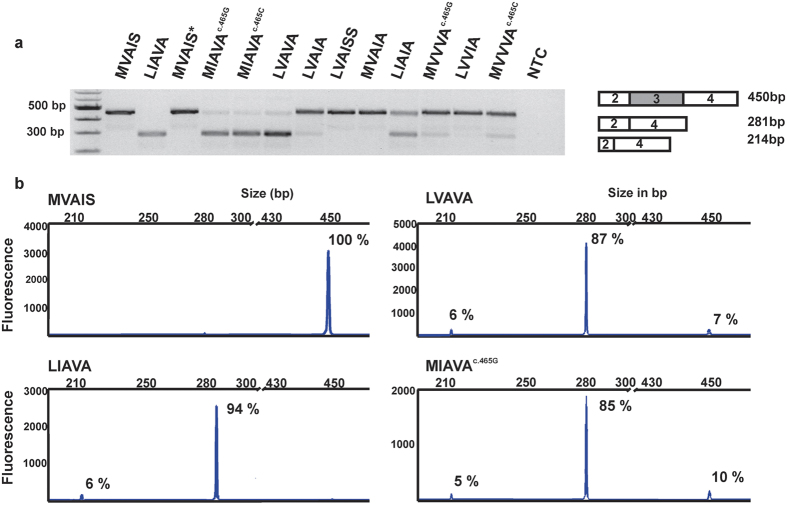
Qualitative and quantitative analysis of RT-PCR products from minigene splicing assays. (**a**) Agarose gel electrophoresis of RT-PCR products obtained with RNA from HEK293 cells transfected with minigene constructs bearing various exon 3 haplotypes. The tested haplotype is given above the corresponding gel lane. A 100 bp ladder size standard was loaded in the leftmost lane. Both lanes ‘MVAIS’ and ‘MVAIS*’ refer to minigenes carrying the control haplotype. ‘MVAIS*’ has a modified Multiple Cloning Site from the prototype construct ‘MVAIS’ (see Supplementary Materials and Methods). NTC: non-template negative control. A scheme on the composition of the RT-PCR products is given on the right. Full length gel picture is presented in Supplementary Fig. S3. (**b**) Examples of capillary electrophoresis and quantitative analysis of fluorescent labeled RT-PCR products of the minigene assay for four different haplotypes (‘MVAIS’, ‘LVAVA’, ‘LIAVA’ and ‘MIAVA^c.465G^’). The fragment size scale is given on the x-axis and fluorescence intensity (in arbitrary units) on the y-axis. Relative amounts of each fragment are given for the corresponding peak as determined by Gene Mapper. The three different sized products correspond to correctly spliced transcripts (450 bp), aberrantly spliced transcripts lacking exon 3 (281 bp), and a minor species of aberrantly spliced products lacking exon 3 and further 72 bp of exon 2 (214 bp).

We analyzed exon 3 transcript processing for a total of twelve different exon 3 haplotypes observed in our patient cohort and the ‘MVAIS’ control haplotype by an established minigene assay^20^. RT-PCR showed three differently spliced products with relative quantities depending on the actual haplotype (Fig. 2a). The 450 bp product is derived from the correctly processed transcript, whereas the two smaller products are derived from aberrantly spliced transcripts either lacking exon 3 (281 bp) or lacking exon 3 and 72 bp of the 3′ end of exon 2 (214 bp), respectively (Fig. 2a). Both aberrant transcripts cause a frame shift leading to a premature termination codon (PTC) in exon 4. To quantify the splicing defect more precisely we performed RT-PCR with a FAM-labeled forward primer and separated the PCR products on a capillary electrophoresis instrument. From the AUC of fluorescence intensity for each fragment we calculated the relative abundance of individual fragments and correctly spliced RT-PCR products (Fig. 2b, Table 2). The control haplotype ‘MVAIS’ yielded no aberrantly spliced products while the ‘LIAVA’ construct resulted in the complete absence of correctly spliced products (Fig. 2b). Three haplotypes, ‘LVAVA’, ‘MIAVA^c.465C^’ and ‘MIAVA^c.465G^’ were found to yield only minor amounts (below ∼10%) of correctly spliced products and were classified as strongly deleterious (+++, Table 2). An intermediate fraction (20–50%) of correctly spliced products was obtained with three further haplotypes, ‘LIAIA’, ‘LIAVS’ and ‘MVAVA’ and were considered intermediate (++) in terms of the magnitude of the splicing defect. The remaining five haplotypes, ‘LVAIA’, ‘LVAISS’, ‘MVAIA’, ‘MVVVA^c.465G^’ and ‘MVVVA^c.465C^’ yielded predominantly (>75%) correctly spliced products. (Fig. 2b, Table 2) and therefore were considered minor defective (+).

### Correlation of *LW/MW* arrays and splicing defects with clinical presentation

*LW/MW* gene cluster genotypes were greatly diverse in our cohort of families with clinical diagnosis of BCM or XLCD. One family had an *LW/MW* gene array made up of a total of four *LW*/*MW* gene copies, four families had three copies, six families had two copies and five families carried a single *LW,* or *LW/MW* hybrid gene.

All families with single gene arrays carried strongly deleterious haplotypes: ‘LIAVA’ in BCM73 and BCM93, and ‘LVAVA’ in another three families (BCM66, BCM112, and BCM194). Clinical data were available for nine subjects from these families (Supplementary Table S1). Except for one very young subject (BCM194–25474), all these subjects had a best corrected visual acuity (BCVA) of ≤0.3, absent or strongly reduced photopic and flicker ERG responses, and impaired colour vision. Seven out of nine subjects had nystagmus and two experienced mild photophobia. Funduscopy revealed minor macular pigmentary irregularities but were dominated by myopia-related alterations such as optic disc crescents and peripapillary atrophy. All but two subjects in this group were highly myopic. Within this group we noted a tendency that subjects with the ‘LIAVA’ haplotype are more severely affected than those with the ‘LVAVA’ haplotype.

The families with multiple *LW/MW* opsin genes were more heterogeneous in terms of genotypes and clinical presentation. Clinical data were available for fifteen subjects from eleven families. Current genotyping technologies cannot determine the actual order of multiple distal *MW* gene copies. Therefore, for ordering of multiple *MW* gene copies we took into account impaired *MW* cone function in the patients along with the positional bias of the opsin gene expression^5^. Three families had identical *MW* gene copies with respect to exon 3 haplotypes whereas BCM142 and BCM72 had different *MW* gene copies (BCM142–21958, BCM72–17075 and BCM72–16874, Table 1). In the latter we assume that the *MW* gene copy harbouring the exon 3 haplotype with the most severe splicing defect occupies the second position in the array. Four families (ZD379, BCM101, BCM126 and BCM72–17075 [BCM72-III:1]) had highly deleterious (+++) haplotypes in all gene copies either being completely (BCM126) or partially isogenic (i.e. all copies with identical haplotypes or two of three gene copies sharing an identical haplotype, respectively). While all subjects in this group were clinically typical for BCM, myopia was rather mild and one was even slightly hyperopic.

The second sub-category of subjects with multiple copies are characterized by a *LW* gene comprising a strongly deleterious exon 3 haplotype and a single or multiple *MW* genes carrying exon 3 haplotypes causing intermediate or minor splicing defects. Although the clinical findings of the seven subjects from five families (BCM51, BCM133, BCM160, ZD314, and ZD547) were rather variable (namely visual acuity, photophobia and nystagmus), we consistently noted myopia, impaired colour vision and reduced but never absent cone or flicker ERG responses in these subjects. Consistent with some preserved central, cone-mediated vision there was at least for the better eye a BCVA of ≥0.5 in four of them.

The remaining subjects are less likely to be explained simply by the splicing defect due to exon 3 haplotypes: BCM98–19713 comprises a ‘LIAIA’ haplotype in the *LW* gene associated with a moderate splicing defect and a common haplotype with minimally compromised splicing in the *MW* gene. BCM142–21958 only had exon 3 haplotypes that result in minor amounts of aberrant transcripts, but carries a novel missense variant, c.556C > T/p.P186S, in exon 3 of the *LW* opsin gene which does not compromise splicing, but may impair folding and/or function of the derived photopigment. Notably, an analogous proline to serine substitution at amino acid position 187 has been reported previously in the *LW* opsin gene of a deuteranope subject^24^.

### Gene conversion results in the deleterious haplotype ‘LIAVA’

**Figure 3:**
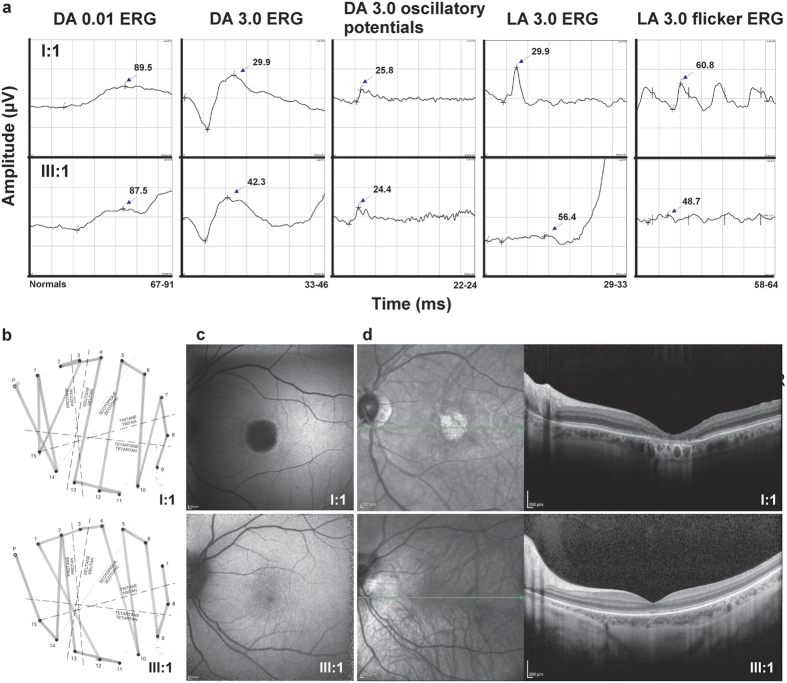
Clinical findings in family BCM72 with strikingly different phenotypes in the grandfather (I:1) and his grandson (III:1). (**a**) Fullfield-ERG with nearly normal responses in BCM72-I:I and not detectable photopic responses in BCM72-III:1. DA: dark-adapted, LA: light-adapted, stimulus strengths: 0.01 or 3.0 cd.s.m^−2^. (**b**) Panel D-15 desaturated with protan defects in BCM72-I:I, and deutan defects in BCM72-III:I. (**c**) Autofluorescence and (**d**) infrared picture (left panel) and OCT (right panel) with macular dystrophy in BCM72-I:I and normal retinal architecture with thinned photoreceptor layer in BCM72-III:I.

We observed a family, BCM72, with rather discordant phenotypes (Fig. 3) between the index subject (BCM72-III:1, Fig. 4a) diagnosed with BCM, and his grandfather (BCM72-I:1, Fig. 4a) who developed macular dystrophy at the age of 40 and presented with a deutan defect. A crucial difference is documented in the cone-derived ERG recordings: Responses in BCM72-I:1 were not impaired, whereas in BCM72-III:1 photopic single-flash and 30 Hz-flicker-responses were extinguished as shown by a flat light-adapted (LA) ERG (Fig. 3a). Rod-derived responses were normal in both cases (Fig. 3a). Subject BCM72-I:1 performed as a deuteranomalous in the Farnsworth Panel D-15 desaturated test while BCM72-III:1 had a majority of confusions along the protan axis (Fig. 3b). A protan-like arrangement of colour discs in the Panel D-15 is frequently observed in BCM subjects^8^,^25^. The index subject’s mother (BCM72-II:2) had normal visual acuity and colour vision but borderline reduced photopic ERG responses. The macular dystrophy of subject BCM72-I:1 is evident in the fundus autofluorescence image of his left eye (Fig. 3c, upper image). OCT of the left eye of both subjects is shown in Fig. 3d. Retinal pigment epithelium and mostly the outer cone photoreceptor layers, namely the cone outer segment termination and the inner segment ellipsoid layers, were severely damaged in the macular area in subject BCM72-I:1, but the retinal thickness is also diminished in subject BCM72-III:1.

**Figure 4:**
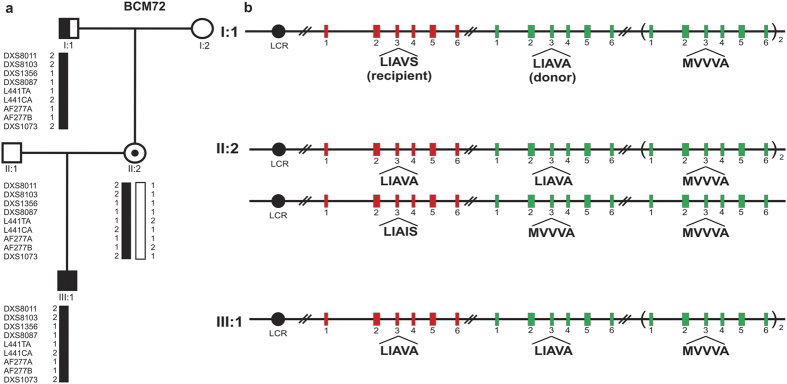
Gene conversion at the *LW/MW* opsin gene cluster in family BCM72. (**a**) Pedigree of family BCM72. Subject BCM72-I:1 presented with macular dystrophy and deuteranopia, subject BCM72-II:2 is an asymptomatic female carrier and subject BCM72-III:1 was diagnosed with BCM. Reconstructed haplotypes based on microsatellite markers encompassing the *LW/MW* gene cluster on Xq28 revealed inheritance of the X-chromosome from the grandfather to the grandson with no evidence for recombination. (**b**) Scheme of the structure of the *LW/MW* gene array and the *LW* and *MW* exon 3 haplotypes in crucial members of the BCM72 family. The *LW/MW* gene array on the transmitted chromosome comprises the locus control region (LCR), a single *LW* gene and three *MW* gene copies. Red and green coloured and numbered boxes represent *LW* exons and *MW* exons, respectively. Exon 3 haplotypes are indicated below the respective exon boxes. For multiple distinct *MW* copies, their most likely order with respect to exon 3 haplotypes is depicted. Note that female subject BCM72-II:2 is heterozygous for *LW* exon 3 haplotypes ‘LIAVA’ and ‘LIAIS’, the latter inherited from her mother. The findings support an intrachromosomal gene conversion event transforming the more ancestral haplotype ‘LIAVS’ into the ‘LIAVA’ haplotype in the germline of subject BCM72-I:1.

**Figure 5:**
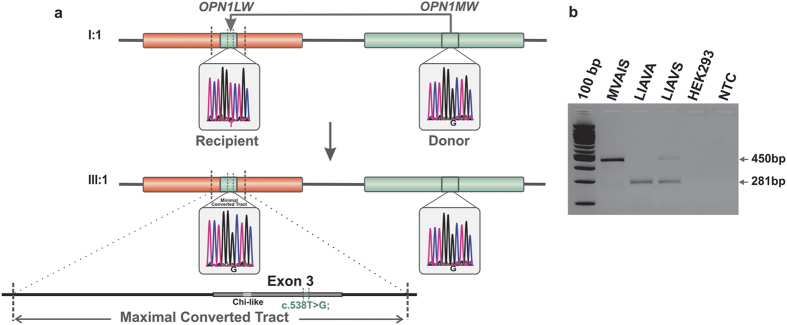
Gene conversion replaces a permissive ‘LIAVS’ haplotype by a strongly deleterious ‘LIAVA’ haplotype in the *LW* gene. (**a**) The gene conversion occurred in a ‘telomeric to centromeric’ direction in the X-chromosome of subject BCM72-I:1 with the ‘LIAVA’ bearing *MW* gene serving as donor and the ancestral ‘LIAVS’ bearing *LW* gene serving as recipient. Below, the X-chromosome of subject BCM72-III:1 presents the product of the gene conversion event with the ‘LIAVA’ haplotype present on both *LW* and the most proximal *MW* as demonstrated by corresponding sequences traces for the c.538T > G variant. Sequencing of cloned *LW* and *MW* gene fragments revealed a maximal converted region of 1,297 bp (c.409 + 950_c.578 + 90conNM_000513.2: c.409 + 950_c.578 + 90) covering exon 3 and flanking intron sequences (see also Supplementary Fig. S2). (**b**) Direct comparison of RT-PCR products from the minigene splicing assays shows a substantial amount of correctly spliced transcripts (450 bp) for the ‘LIAVS’ exon 3 haplotype, whereas such products are undetectable for the ‘LIAVA’ haplotype. Full length gel picture is presented in Supplementary Fig. S4.

Genotyping of the grandson BCM72-III:1 revealed an intact *LW/MW* gene array with a proximal *LW* gene and three copies of the *MW* gene (Fig. 4b). Sequence analysis showed a ‘LIAVA’ exon 3 haplotype for the *LW* gene and ‘LIAVA’ and ‘MVVVA^c.465C^’ for the *MW* gene copies. No further mutations were found in other exons (including exon 6, which is not covered by the LD-PCRs) of the opsin genes of the affected subjects. Considering the expression bias of the opsin gene cluster, and the BCM phenotype of the subject, we reasoned that the ‘LIAVA’-bearing *MW* gene copy occupies the first position downstream of the *LW* gene. Surprisingly, the same downstream *MW* haplotypes but a distinct *LW* exon 3 haplotype ‘LIAVS’ were found in the grandfather BCM72-I:1. Applying the minigene splicing assay we found that the ‘LIAVS’ haplotype resulted in an intermediate splicing defect with still approximately 20% correctly spliced transcripts (Fig. 5b).

Segregation analysis with microsatellite markers flanking the *LW/MW* gene cluster supported the transmission of the Xq28 segment from grandfather to grandson without evidence for recombination (Fig. 4 and Supplementary Fig. S1), suggesting a *de novo* gene conversion in the *LW/MW* gene cluster in this family. We further explored whether the gene conversion event has occurred in *trans* between X-chromosomes (in the mother’s germline), or in *cis* between *LW* and *MW* gene copies in either the grandfather’s or the mother’s germline. Genotyping of the mother BCM72-II:2 revealed a common ‘LIAIS’ exon 3 haplotype in one of her proximal *LW* genes and the ‘LIAVA’-bearing haplotype in the other *LW* gene copy (Fig. 4b). This finding strongly supports transmission of the ‘LIAVA’-bearing *LW* gene from her father (BCM72-I:1) and from BCM72-II:2 to her offspring and thereby asserts the occurrence of the gene conversion in the grandfather’s germline as a *de novo* mutation. To define the extent of this intrachromosomal event we sequenced exon 3 and the flanking introns of the putative gene conversion donor (‘LIAVA’-bearing *MW* gene copy) and recipient gene copy (‘LIAVS’-bearing *LW* gene copy) in BCM72-I:1 and its product (‘LIAVA’-bearing *LW* gene copy) in both BCM72-II:2 and BCM72-III:1. By comparative sequencing we narrowed down the size of the maximal converted sequence in the recipient *LW* to a region of 1,297 bp (c.409 + 950_578 + 90conNM_000513.2:c.409 + 950_578 + 90), which is delimited by the discriminative SNP rs3788802 (c.409 + 949 G > A) in intron 2 and rs369018729 (c.578 + 91 G > A) in intron 3, and includes the discriminating variant c.538 T > G/p.S180A/rs949431 in exon 3 (Fig. 4a and Supplementary Fig. S2).

## Discussion

### Splicing defects caused by rare *LW/MW* exon 3 haplotypes

The presence of rare combinations (foremost ‘LIAVA’) of otherwise common coding variants in exon 3 of the *LW/MW* genes in subjects with X-linked colour vision or cone dysfunction disorders has been noted in several publications^9^,^18^,^24^. Ueyama and colleagues were the first who showed that such rare combinations of variants induce a splicing defect and hence, explain the protan defect in probands with otherwise normal *LW* gene sequence^20^. Lately – and during the course of our study – Gardner *et al*. reported *LW/MW* gene splicing defects as underlying disease mechanism in nine BCM/XLCD families carrying exon 3 “interchange haplotypes”^21^. These patients had only a single *LW/MW* gene copy carrying an exon 3 haplotype inducing a splicing defect or multiple opsin gene copies, in which at least the two most proximal carry such haplotypes. In this study we could corroborate their findings to a broader extent by including a large series of subjects with BCM or XLCD (24 affected subjects from 16 families). We observed an extensive diversity in terms of structure and composition of the *LW/MW* gene cluster and exon 3 haplotypes. Besides, we explored the functional consequences of twelve exon 3 haplotyes on RNA processing by means of heterologous minigene assays and took advantage of fluorescence-based capillary electrophoresis of RT-PCR products for accurate relative quantification of the proportions of differentially spliced transcripts. Most of the investigated haplotypes resulted in a substantial fraction of mis-spliced transcript with two main aberrant mRNA species that result in a PTC in exon 4. Such transcripts presumably elicit nonsense-mediated mRNA decay^26^. Accurate quantification of the relative amounts of the different species disclosed clearly distinct ratios of correctly and aberrantly spliced transcripts depending on the haplotype. ‘LIAVA’ as the extreme led to complete exon skipping. The two related haplotypes ‘MIAVA^c.465C^’ and ‘MIAVA^c.465G^’, as well as ‘LVAVA’, yielded high levels of aberrantly spliced transcripts but still detectable amounts of correctly spliced transcripts. Notably, we observed the latter haplotype being associated with a less severe clinical phenotype in comparison with patients harbouring ‘LIAVA’. Three further haplotypes, ‘LIAVS’, ‘LIAIA’ and ‘MVAVA’ have an intermediate effect in terms of aberrant splicing (20–50% of correctly spliced opsin), whereas the remaining six haplotypes confer only minor splicing defects. The (patho-)physiological consequences of such intermediate or minor defects on disease expression or colour in general still needs to be explored.

The consequences of genotypes that comprise highly deleterious exon 3 haplotypes in the relevant, most proximal gene copies may approximate the situation of a full *null* mutation like patients with larger deletions at the *LW/MW* gene cluster^9^,^12^,^13^,^14^. Less straightforward are cases with multiple gene copies combining copies with highly deleterious exon 3 haplotypes and copies with haplotypes causing intermediate splicing defects (e.g. *LW*: ‘LVAVA’+*MW*: ‘MVAVA’ as in ZD547). The diversity of genotypes and quantitative difference in the magnitude of the splicing defect caused by different haplotypes is reflected by the variability in clinical presentations ranging from a BCM typical phenotype to cone disorders with considerably preserved cone function and rather good visual acuity. This clinical variability is in line with previous reports on subjects with BCM, “atypical BCM”, or XLCD who carry rare *LW/MW* exon 3 haplotypes^9^,^16^,^17^,^18^.

Since accurate quantification of the different spliced forms is lacking in prior studies, we could not directly compare their results at the quantitative level. However, a qualitative comparison points out some differences. While in this study as well as in the study of Gardner and colleagues^21^ the three distinct transcript species were observed, the correctly spliced form, an exon 3-skipped transcript and an additional aberrant transcript, the nature of the latter one seems to differ. This third minor species is produced for instance from the ‘LVAVA’ and ‘MIAVA’ haplotypes (and from ‘LIAVA’ in our study) which in both studies is a result of internal exon 2 splicing but involves different acceptor sites in intron 3 (in the study of Gardner and colleagues) and at the correct intron 3/exon 4 junction (in this study), respectively. Microheterogeneity in terms of minor aberrant splicing products have not only been observed in many heterologous minigene assays but also in native tissue of subjects carrying splicing-inducing mutations^27^ and may be influenced by technical parameters.

SROOGLE^28^ predicted several Splicing Regulatory Sequences (SRSs) to be disrupted and/or created when exon 3 SNPs from deleterious haplotypes were interchanged. In fact, every other SNP within the haplotype disrupts or creates at least one SRS. Half of the overall putative Exonic Enhancers of Splicing (EESs) overlapping the eight SNPs were predicted to be disrupted by ‘LIAVA’. For instance, the c.521C > T substitution is foreseen to create a novel EES, which is consistent with the increased correctly spliced transcripts abundance *in vitro* (Table 2, haplotype 9–10). Comparison of closely related haplotypes indicates that the c.532A/p.178Ile allele confers “protection”: for instance ‘LVAVA’ yielded about 7% of correctly spliced transcripts whereas ‘LVAIA’ about 80% (Table 2, haplotype 4–5). These patterns may suggest the presence of motifs that exert combined enhancer and silencer effects to a certain extent as described in *SMN1* and *CFTR* genes^29^.

### Gene conversion as a mechanism leading to a pathogenic haplotype in the cone opsin array

We here report a *de novo* gene conversion event in the *LW/MW* gene cluster which we proved in the lineage of a single family (BCM72). This rare instance further enabled us (i) to discriminate between inter- and intrachromosomal event, (ii) to differentiate between its occurrence in either the male versus the female germline, (iii) to define the directionality of the event (from *LW* to *MW* or *vice versa*), (iv) to determine the actual sequence homology between the donor and recipient prior to the gene conversion event and finally (v) to refine the extent of the converted sequence. These findings are novel since no other *de novo* gene conversion events in a parent-offspring transmission have been reported for the *LW/MW* gene cluster so far.

Our study is in congruency with the population-based evidence for gene conversion in the opsin genes as described by Verrelli and Tishkoff^23^. Gene conversion together with selective forces are proposed to have an influence on the observed variation in *LW*/*MW* genes, as mainly advantageous polymorphisms have been spread^6^,^23^. However, beyond the evolutionary perspective, gene conversion in the *LW/MW* gene cluster may also lead to the occurrence of deleterious genotypes that are associated with colour vision deficiencies, XLCD or BCM.

Signatures of gene conversion in the *LW/MW* gene cluster at the level of individual chromosomes have so far been proposed specifically in the context of BCM subjects with rare point mutations present in all copies of the *LW/MW* gene cluster^13^,^30^, yet the gene conversion event could not be directly pinpointed, hindering a full characterization. For instance, Gardner and colleagues reported a BCM family in which the c. 529T > C/p. W177R missense mutation in exon 3 was observed in both *LW* and *MW* genes^13^. Albeit a gene conversion event was deduced based on the observation that both genes carry this unique mutation and share a block of SNPs in exon 3, occurrence of the gene conversion event (i.e. a family member with the ancestral genotype prior to the event) could not be observed in this family.

Different from these previous reports, we herein were able to study for the first time a gene conversion event at the *LW/MW* gene cluster within the lineage of a single pedigree (i.e. the genotypes prior to and following the gene conversion event) and, importantly, to associate these two different genotypes to their cognate phenotypes. Furthermore, we could correlate these differences at the phenotypic level prior and post-gene conversion with a quantitative difference in the *in vitro* levels of correctly spliced *LW* gene transcripts that results from the distinct exon 3 haplotypes prior and post gene conversion. While ‘LIAVA’ has been previously assessed for splicing^20^, the ancestral prior gene conversion ‘LIAVS’ haplotype has been reported in the literature^18^ but has – to our knowledge – never been tested for splicing before.

With respect to directionality of the gene conversion, we could demonstrate in our family that the *MW* copy acted as donor and *LW* copy served as recipient gene copy. The same ‘telomeric to centromeric’ direction has been proposed for the “spreading” mutation cases^13^,^30^. Seemingly, the position of a recombination hotspot has an influence over the directionality of gene conversion^31^. The donor *MW* copy carries the haplotype ‘LIAVA’, which most likely occupies the first position downstream of the *LW* gene, explaining the deutan defect in BCM72-I:1. The *LW* gene of this subject carries the exon 3 haplotype ‘LIAVS’, which only differs from ‘LIAVA’ by a single SNP (c.538G > T/p.S180A). Compared to the latter haplotype which results in a fully penetrant splicing defect, the ‘LIAVS’ haplotype produces 20% of correctly spliced transcripts which is perfectly compatible with the much milder phenotype of the grandfather (BCM72-I:1). In contrast, the grandson (BCM72-III:1) presenting with BCM has the two first *LW/MW* copies carrying the fully deleterious haplotype ‘LIAVA’, consistent with his BCM phenotype. The ERG responses were the most informative clinical feature that documents the phenotypic differences between BCM72-I:1 and BCM72-III:1. The ‘LIAVS’ haplotype has been reported before in affected males of two families with X-linked cone-dominated phenotypes^18^, who compared to the ‘LIAVA’-carrying individuals from an independent family seemed to have a better visual performance. One of the ‘LIAVS’-carrying individuals presented with evidence of maculopathy, similarly to BCM72-I:1 here, however, BCM72-I:1 developed this maculopathy later in life.

Given the sequence identity between *LW* and *MW*, we could only define the outermost borders of the gene conversion event in intron 2 and intron 3 (Fig. 5a) setting a maximal size of about 1,300 bp which is beyond the average size estimates for converted tracts in humans^32^. Significantly reduced linkage disequilibrium of variants in a region of about 400 bp covering *LW* exon 3 and flanking intronic sequences supported the presence of a hotspot for gene conversion at this region^23^ centering around a *Chi*-like sequence element^22^. Although gene conversion at a certain site is expected to be a rare event, recent single cell analysis technologies may allow to actually determining the frequency and pattern of such events at the *LM/MW* locus in human sperm cells^33^.

While formally a *de novo* point mutation could be an alternative explanation, we reason that a *de novo* gene conversion is the most likely event since (i) the very same c.538T> G variant is present in the downstream *MW* gene copy, (ii) the c.538T > G is a common variant frequently observed in *LW* genes^22^, (iii) population studies have revealed strong evidence for frequent gene conversion between *LW* and *MW* genes^23^,^34^, and (iv) it has been estimated that the *de novo* rate of gene conversion between paralogous loci in the human genome is approximately 100-fold higher than the *de novo* point mutation rate^35^,^36^,^37^,^38^.

The current study, not only supports the predictions of population genetics studies and “spreading” mutation cases on gene conversion in the opsin genes, but also, adds direct evidence on how gene conversion events may arise spontaneously in the germline having an important impact on human disease. Moreover, this type of mutations might be overlooked by employing next-generation sequencing technologies which complicates its identification as well as its prediction.

In conclusion, our study confirms that *LW/MW* gene array genotypes bearing certain rare exon 3 haplotypes cause BCM and XLCD in a large patient cohort and explains the deleterious functional effect of these haplotypes. Reliable quantitative analysis of splice products has been implemented and revealed that the relative proportions of correctly and aberrantly spliced transcripts obtained for the twelve different haplotypes cluster in three main categories. Lastly, we traced back the origin of the strongly deleterious ‘LIAVA’ haplotype in a BCM subject. This haplotype was inherited from his mother and, in all likelihood arose as a result of a *de novo* intrachromosomal gene conversion event from an ancestral ‘LIAVS’ in the grandfather’s germline and explains the phenotypic transformation from deuteranopia in the grandfather to BCM in his grandson.

## Additional Information

**How to cite this article**: Buena-Atienza, E. *et al*. *De novo* intrachromosomal gene conversion from *OPN1MW* to *OPN1LW* in the male germline results in Blue Cone Monochromacy. *Sci. Rep.* **6**, 28253; doi: 10.1038/srep28253 (2016).

## Supplementary Material

Supplementary Information

## Figures and Tables

**Table 1:** *LW/MW* gene cluster composition and *LW* and *MW* exon 3 haplotypes in BCM and XLCD families analysed in this study.

Index subjects	Exon 3					Clinical diagnosis
	*LW*		*MW*			
	Haplotype	p.	Haplotype	p.	n^d^	
ZD379-19194	1	LIAVA	2	MIAVA^c^^.465C^	2	BCM
BCM101-19818	1	LIAVA	3	MIAVA^c^^.465G^	2	BCM
BCM51-12359	1	LIAVA	9	MVVVA^c^^.465G^	2	XLCD
BCM160-23130	1	LIAVA	9	MVVVA^c^^.465G^	1	BCM
ZD314-18057	1	LIAVA	10	MVVVA^c^^.465C^	1	XLCD
BCM126-20616	4	LVAVA	4	LVAVA	1	BCM
BCM133-20961	4	LVAVA	5	LVAIA	1	BCM
ZD547-4544	4	LVAVA	12	MVAVA	1	XLCD
BCM98-19713	8	LIAIA	7	MVAIA	1	XLCD
BCM142-21958	6	LVAIS^c^	5, 7	LVAIA, MVAIA	2	BCM
BCM72-17075^a^	1	LIAVA	1, 10	LIAVA, MVVVA^c.465C^	3	BCM
BCM72-16874^a^	11	LIAVS	1, 10	LIAVA, MVVVA^c^^.465C^	3	Deutan/Macular Dystrophy
BCM73-16953^b^	1	LIAVA	–	–	0	BCM/XLCD
BCM93-19164	1	LIAVA	–	–	0	XLCD
BCM66-16407	4	LVAVA	–	–	0	BCM
BCM112-23518^b^	4	LVAVA	–	–	0	CRD^e^
BCM194-25474	4	LVAVA	–	–	0	BCM

^a^Affected subjects from the same family with distinct genotypes.

^b^Subjects harbouring single *LW/MW* hybrid genes.

^c^This haplotype LVAIS includes an additional missense variant (c.556C > T/p.P186S; RefSeq: NM_020061.5) in exon 3 of the *LW* gene.

^d^Number of *MW* gene copies deduced from qPCR.

^e^Cone-Rod Dystrophy due to additionally impaired rod function (see Supplementary Table S1).

**Table 2:** Relative quantification of the proportion of correctly spliced transcripts for different exon 3 haplotypes.

	Exon 3 (p.)^a^	Subjects^b^	%Correctlyspliced^c^	SD^d^	Splicingdefect^e^	PopulationFrequency(*LW*/*MW*)^f^
Control Haplotype^g^	MVAIS	−	100	−	−	0.027/0.008
Haplotype 1	LIAVA	9	n.d.	−	+++	0.0/0.0
Haplotype 2	MIAVA^**c**^^.465G^	1	10.41	1.45	+++	0.0/0.0
Haplotype 3	MIAVA^**c**^^.465C^	1	8.78	3.19	+++	0.0/0.0
Haplotype 4	LVAVA	6	6.71	0.27	+++	0.0/0.0
Haplotype 5	LVAIA	2	79.40	0.97	++	0.23/0.033
Haplotype 6	LVAIS^h^	1	98.73	1.10	+	0.0/0.0
Haplotype 7	MVAIA	1	97.62	0.19	+	0.094/0.6
Haplotype 8	LIAIA	1	40.75	0.23	++	0.0/0.0
Haplotype 9	MVVVA^**c**^^.465G^	2	80.07	0.35	+	0.0/0.025
Haplotype 10	MVVVA^**c**^^.465C^	3	75.59	0.65	+	0.027/0.21
Haplotype 11	LIAVS	1	20.3	0.0	++	0.0/0.0
Haplotype 12	MVAVA	1	53.0	0.0	++	0.013/0.016

^a^Exon 3 haplotypes referring to the encoded amino acid combination.

^b^Number of subjects (index) carrying this haplotype.

^c^Proportion of RT-PCR products from correctly spliced transcripts (450 bp).

^d^Standard deviation (SD), calculated from technical triplicates.

^e^Relevance of the splicing defect (−, no splice defect; +, more than 75% correctly spliced product; ++, 20–50% of correctly spliced product; +++, below ~10% of correctly spliced product ).

^f^Frequency of haplotypes in the population as reported by Winderickx *et al*.^22^.

^g^Control haplotype as reported by Ueyama *et al*.^20^.

^h^This haplotype LVAIS includes an additional missense variant (c.556 C > T/p.P186S; RefSeq: NM_020061.5) in exon 3 of the *LW* gene.
