# Supplementary material for: De novo intrachromosomal gene conversion from OPN1MW to OPN1LW in the male germline results in Blue Cone Monochromacy
Source: Sci Rep. 2016 Jun 24;6:28253. doi: 10.1038/srep28253 (PMC4919619; doi:10.1038/srep28253)
Supplement: Supplementary Information [file srep28253-s1.pdf]

Supplementary Information

De novo intrachromosomal gene conversion from *OPN1MW* to *OPN1LW* in the male germline results in Blue Cone Monochromacy

Authors

Elena Buena-Atienza [1], Klaus Rüther [2, 3], Britta Baumann [1], Richard Bergholz[2], David Birch[4], Elfride De Baere [5], Helene Dollfus [6], Marie T. Greally [7], Peter Gustavsson [8], Christian P. Hamel [9], John R. Heckenlively [10], Bart P. Leroy [5], Astrid S. Plomp [11], Jan Willem R. Pott [12], Katherine Rose [13], Thomas Rosenberg [14], Zornitza Stark [15], Joke B. G. M. Verheij [16], Richard Weleber [17], Ditta Zobor [1], Nicole Weisschuh [1], Susanne Kohl [1], Bernd Wissinger [1*]

Supplementary Materials and Methods

Patient recruitment and clinical evaluation

The study was performed in compliance with the tenets of the WMA Declaration of Helsinki. Study participants diagnosed either with BCM or XLCD were recruited *ad hoc* at different centers specialized in inherited retinal diseases during routine clinical diagnostics. All participants gave written informed consent – approved by the respective local research and ethical review boards - for participation in the study for which blood or DNA samples were sent to Tuebingen for genetic analysis. Procedures of the genetic analysis were approved by the Ethics Committee of the Medical Faculty, Eberhard-Karls University Tuebingen. Patients underwent basic ophthalmologic examination at the different clinical centers which included evaluation of ocular motility and slit lamp examination of the anterior segment, visual acuity (VA), refraction, colour vision and visual field testing, assessment of retinal morphology applying imaging technologies such as funduscopy, optical coherence tomography (OCT), autofluorescence imaging, and ERG recordings under scotopic and photopic conditions.

Genotyping of the *MW/LW* gene cluster

Genomic DNA was isolated from blood samples according to standard procedures. The structure and integrity of the *LW*/*MW* gene cluster (e.g. single *versus* multiple opsin gene copies, presence of hybrid genes) on the X-chromosome was investigated by a two-step genotyping protocol. First, we used a protocol that screens for mutations most commonly found in BCM/XLCD patients and specifically tests for large genomic deletions at the *OPN1LW*/*OPN1MW* gene cluster and the upstream locus control region (LCR), the presence of single or multiple opsin gene copies, and the presence or absence of the common point mutation c.607T>C, p.C203R. Briefly, duplex PCRs including primers for the tested fragment and an autosomal control fragment were performed for: 1) a sequence defined as the common overlap of known deletions of the LCR (Nathans et al., 1989, 1993); 2) the *OPN1LW* promoter; and 3) the *OPN1MW* promoter to test for large genomic deletions and the presence of single or multiple opsin gene copies. In addition, two PCR fragments for exons 4 and 5, respectively, for both *OPN1LW* and *OPN1MW* were amplified and digested with B*stUI* and R*saI*, respectively. The restriction fragment length polymorphism (RFLP) in exon 4 via B*stUI* analysed for the c.607T>C p.C203R mutation (RFLP positive cases are further validated by Sanger sequencing), while the RFLP in exon 5 via R*saI* addressed the dimorphism at amino acid position 277 – p.Y277 being specific for the *OPN1LW* gene, and p.F277 for the *OPN1MW* gene. The second screening step analyzed for other deleterious point mutations or haplotypes for those subjects either single or multiple structurally intact gene copies by PCR and Sanger-sequencing. Specific long distance PCRs (LD-PCRs) were performed with primers LCR1F (5'-CACCCTTCTGCAAGAGTGTGGG-3') and RGCP2-5R (5'-GCGGACTAGTGATCTGCTGATGGTGTTGCTTA-3') for the proximal gene copy, and E1G-F (5'-GAGTACAGGTATTTGCCACTAAGC-3') and RGCP2-5R for distal gene copies. LD-PCR products were

directly sequenced with internal primers or re-amplified with exon-specific primers prior to sequencing. PCR products were purified by ExoSAP-IT treatment (Affymetrix, Santa Clara, CA) and cycle-sequenced applying BigDye Terminator V1.1 chemistry (Life Technologies, Darmstadt, Germany). For the determination of exon 3 haplotypes we amplified a 400 bp fragment using primers BCM-Ex3-F (5'-TGGTGGAAAGAAAGATGTCG-3') and BCM-Ex3-R (5'-GCCCAGAGAAAGGAAGTGATT-3') that was subsequently sequenced directly or after an intermitting cloning step if multiple mixed base positions were observed.

For subjects with multiple gene copies, the total number of *LW/MW* opsin genes was determined by means of real-time quantitative PCR (qPCR) with genomic DNA as template. We used two different TaqMan assays that target different parts of the *LW/MW* genes: the HS_01912094 assay (Life Technologies) targeting exon 6, and a custom-designed TaqMan assay (employing RGCP_TQF [5'-CCCAACAGAAAGCTGAAAGC-3'] as forward and RGCP_TQR [5'-GTGCAAAACTTTCGGATTGG-3'] as reverse primers, respectively, and RGCP_TQP [5'-CAGCCCGAGTCCTGCCATTGG-3'] with 5'-FAM and 3'-BHQ1 modifications as probe primer) targeting a common segment of intron 1. In parallel, we performed qPCR reactions for a human genome single copy reference sequence (RNaseP TaqMan Copy Number Reference Assay; Life Technologies). We used a series of male controls with defined *LW/MW* copy number [n=1-6; (4)] to generate a copy number dependent ΔCt calibration curve. All three assays were performed in triplicate for each sample. Obtained ΔCt values were used to infer copy numbers from the calibration curves.

Minigene Constructs

A *Hind*III site in the polylinker of the prototype construct was eliminated for the present study by means of inverse PCR with primers *Not*I-Inv3-Rv (5'-AAAAAAGCGGCCGCTGACGGTTCACTAAACGAGCTCT-3') and *Not*I-Inv3-Fw (5'-AAAAAAGCGGCCGCCGGATCCGGTACCATGGCCCAGCA-3'), followed by digestion with *Not*I and re-circulation. Derived minigenes with novel exon 3 haplotypes were generated by replacing a 279 bp *Hind*III-*Afl*II fragment with a homologous fragment from defined subject obtained through PCR amplification with primers BCM-Ex3-r and BCM-Ex3-f and digestion with *Hind*III and *Afl*II. Alternatively, PCR products were first cloned in pCR2.1 (TA Cloning Kit, Invitrogen, Life technologies) before excision of the *Hind*III-*Afl*II fragment and replacement cloning into the revised prototype construct. In vitro mutagenesis with primers IVM-Ex3-174t-Fw (5'-TGGCCATCGTGGGCATTGTCTTCTCCTGGATCTG-3') and IVM-Ex3-174t-Rv (5'- CAGATCCAGGAGAAGACAATGCCCACGATGGCCA-3') was performed to obtain the haplotype 'LVVIA'. All constructs were verified by Sanger sequencing.

Transfection and RNA extraction

HEK293 cells were routinely maintained in Dulbecco´s modified Eagle´s medium, DMEM (Gibco, Life Technologies) supplemented with 10% fetal bovine serum (FBS; Gibco, Life Technologies), Penicillin-Streptomycin (Sigma-Aldrich Chemie GmbH) at 100 µg/ml, and Amphotericin B (Biochrom GmbH) at 2.5 µg/ml. Cells were seeded in 6-well plates in DMEM with 10% FBS and the following day, at 80-90% confluency, cells were transfected with 4 µg DNA of the minigene construct using 20 µl Lipofectamine 2000 per well and OptiMEM® supplemented with GlutaMAX™ (Life Technologies) as diluents and medium. After 6 h incubation, cells were harvested by trypsinization with 0.05% Trypsine-EDTA (Gibco, Life Technologies), centrifuged at 1500 rpm for 5 min and transferred to a 6 cm dish with DMEM supplemented with 10% FCS and antibiotics. 24 h post-transfection, cells were lysed and total RNA was extracted applying the peqGOLD Total RNA Kit (PEQLAB Biotechnologie GmbH).

RT-PCR and relative quantification

First strand cDNA synthesis was performed using 2 µg of total RNA and random hexamer primers, according to manufacturer's instructions (Transcriptor First Strand cDNA Synthesis Kit, Roche). Subsequent PCR was performed with a 5' FAM (6-carboxyfluorescein) labeled forward primer, FEO35: 5'-ACCATGAAGTTCAAGAAGCT-3', and O4-104-Rv:5'-AGCAGGTGACCATGAGGA-3' as reverse primer and using the QIAGEN Multiplex PCR Kit reagent chemistry (Qiagen) including 1/10 volume of Q-solution. Cycling conditions were 95°C for 15s, 40 cycles of 94°C for 30s, 60°C for 90s and 72°C for 45 to 60s, and a final extension at 72°C for 7 min. PCR products were separated by electrophoresis on a 2% agarose gel. FAM-labeled RT-PCR products were diluted 1:10 in water and mixed with 1 µl of GeneScan ROX500 size standard (Life Technologies) and 8 µl of Hi-Di Formamide (Life Technologies) in a total volume of 10 µl. Mixes were separated by capillary electrophoresis on an ABI 3130XL Genetic Analyzer instrument (Life Technologies). The area-under-the-curve (AUC) was calculated with GeneMapper 5 (Life Technologies) software. Ratios of splicing products were determined as the AUC for individual peaks divided by the sum of AUC of all differentially spliced products.

Microsatellite analysis

Centromeric (DXS8011, DXS8103, DXS1356, DXS8087) and telomeric (L441TA, L441CA, AF277A, AF277B and DXS1073) markers to the *LW/MW* cluster were used to genotype the three BCM72 family members (see Figure. 4A, Supplementary Table S2 and Supplementary Fig. S1). Primers were labeled either with TET, HEX or FAM. Standard PCR reactions were performed with 50-100 ng of genomic DNA. PCR products were diluted 2-10 fold with water, mixed with GeneScan ROX500 size standard and Hi-Di Formamide, and separated by capillary electrophoresis. GeneMapper 5 Software was used for allele calling. Markers for which the mother BCM72-II:1 was heterozygous were used to reconstruct haplotypes.

Mapping of the gene conversion event

LD-PCRs were performed for all three members of BCM72 using primers LCR1F and RGCP2-5R for the proximal gene copy, and E1G-F and RGCP2-5 for the distal copies. Upon *Avr*II-*Kpn*I digestion, the resulting 7494 bp and 6211 bp fragments from *LW* (intron 1 to intron 5) and *MW* (intron 1 to intron 4) respectively, were purified with CHROMA SPIN+TE-1000 columns (Takara Bio Europe) and ligated to *Xba*I-*Kpn*I double-digested pGEM3Zf(+). We selected two independent clones of the following gene copies for further analysis: (1) *LW*-derived clones bearing the 'LIAVA' haplotype from subjects BCM72-II:1 and BCM72-III:1, (2) *MW*-derived clones bearing the 'LIAVA' haplotype from subject BCM72-I:1, and (3) *LW*-derived clones bearing the 'LIAVS' haplotype from subject BCM72-I:1. We sequenced exon 3 and flanking introns in these clones by means of primer walking.

Bioinformatic predictions and reference sequences

In silico splicing predictions were performed with SROOGLE (http://sroogle.tau.ac.il/) (28). NG_009105.2, NG_011606.1, NM_020061.5 and NM_000513.2 as reference sequences of the human *OPN1LW* and *OPN1MW* genes (NG) and mRNA transcripts (NM), respectively (NCBI database). All four reference sequences carry the exon 3 haplotype 'MVAIA'.

Supplementary Tables and Figures

Supplementary Table S1. Compilation of patients' demographics, clinical findings and diagnosis, and genotypes.

Subject code	Age at (last) clinical examination	VA: OD OS	Refraction: OD OS	Fundus	Color vision	ERG	Photophobia	Nystagmus	Progression	Clinical diagnosis	Exon 3 Genotype(s)
**Single *LW* or *LW/MW* Hybrid Gene Array**											
BCM73-16953	51 years	0.15<br>0.15	-12<br>-12	Peripapillary atrophy, slight pigmentary changes in macula	Impaired,<br>OD: blue-yellow axis<br>OS: no specific axis	Photopic / 30 Hz flicker: no response<br>Scotopic: normal	No	Yes	No	BCM	Single LW/MW hybrid; 'LIAVA'
BCM73-17481	14 years	0.05<br>0.05	-14<br>-14	Peripapillary atrophy, slight pigmentary changes in macula and blond appearance of fundus	ND	Photopic / 30 Hz flicker: no response<br>Scotopic: normal	No	Yes	No	BCM	Single LW/MW hybrid; 'LIAVA'
BCM73-20537	3 years	0.2<br>0.2	-4.0(-1.75x30°)<br>-4.0(-2.0x170°)	Peripapillary atrophy, blond appearance of fundus, slight pigmentary changes in macula	ND	Photopic: severely reduced<br>Scotopic: normal	No	Yes	No	XLCD	Single LW/MW hybrid; 'LIAVA'
BCM73-20770	3 months	0.2<br>0.2	-8.0(-1.25x30°)<br>-9.0(-1.50x155°)	Blond appearance of fundus	ND	ND	No	Yes	No	BCM	Single LW/MW hybrid; 'LIAVA'
BCM93-19164	14 years	0.3<br>0.3	-5<br>-3	Myopic optic nerve, peripapillar choroid atrophy, retinal atrophy at posterior pole	Impaired	Photopic: no response<br>Scotopic: normal	No	Yes	No	XLCD	Single MW gene; 'LIAVA'
BCM66-16407	41 years	0.3<br>0.3	-13<br>-14	Peripapillary atrophy, myopic choroidosis	Extreme red-green defect; Berson test positive for BCM	Photopic: absent<br>Scotopic: normal	Very mild	No	No	BCM	Single LW gene: 'LVAVA'
BCM112-23518	14 years	0.16<br>0.16	-24<br>-23	Peripapillar choroid atrophy, thinning of RPE at posterior pole	Protan defect, consistent with incomplete achromatopsia	Photopic: no response<br>Scotopic: severely reduced	No	Yes	Slowly progressive	CRD	Single LW/MW hybrid: 'LVAVA'
BCM112-22852	6 years	0.3<br>0.3	-9<br>-9.5	Diminished foveal reflexes	Protan defect, consistent with incomplete achromatopsia	Photopic: severely reduced<br>Scotopic: slightly reduced	No	Yes	Slowly progressive	CRD	Single LW/MW hybrid: 'LVAVA'
BCM194-25474	5 years	0.5<br>0.5	-16.00(-1.75x35°)<br>-16.25(-1.00x35°)	Temporal optic nerve atrophy, no true fovea reflex, global hypo-pigmented fundus	Normal children's Ishihara test in both eyes.	Photopic: strongly reduced,<br>30Hz Flicker: reduced and delayed<br>Scotopic: normal	Mild	No	Progressive myopia	BCM	Single LW gene: 'LVAVA'

Supplementary Table S1 (continued) - Compilation of patients' demographics, clinical data and diagnosis, and genotypes.

Subject code	Age at (last) clinical examination	VA:<br>OD<br>OS	Refraction:<br>OD<br>OS	Fundus	Color vision	ERG	Photophobia	Nystagmus	Progression	Clinical diagnosis	Exon 3 Genotype(s)
**Multigene LW/MW Gene Array**											
ZD379-19194	12 years	0.16<br>0.16	ND	Mild optic disc pallor	ND	Photopic / 30 Hz flicker: strongly reduced<br>Scotopic: normal	ND	Yes	No	BCM	Multigene (n=3)<br>LW(prox.): 'LIAVA'<br>MW(distal): 'MIAVA$^{c.465C}$'
ZD379-19195	7 years	0.16<br>0.16	+1<br>+1	Peripapillary atrophy and myopic fundus; some macular RPE mottling	ND	Photopic / 30 Hz flicker: strongly reduced<br>Scotopic: normal	ND	ND	ND	BCM	Multigene (n=3)<br>LW(prox.):' LIAVA'<br>MW(distal): 'MIAVA$^{c.465C}$'
BCM101-19818	3 years	0.1<br>0.1	-6<br>-6	Normal	Impaired	Photopic: no response<br>Scotopic: normal	Yes	Yes	No	BCM	Multigene (n=3)<br>LW(prox.): 'LIAVA';<br>MW(distal): 'MIAVA$^{c.465G}$'
BCM126-20616	41 years	0.25<br>0.20	ND	Temporal optic atrophy, no bone spicules or pigment deposits	Impaired;<br>F-M: several confusions along deutan axis	Photopic / 30 Hz flicker: strongly reduced and delayed<br>Scotopic: normal	Yes	ND	Yes	BCM	Multigene (n=2)<br>LW(prox.): 'LVAVA'<br>MW(distal): 'LVAVA'
BCM72-17075 (BCM72-III:1)	12 years	0.1<br>0.1	+0.5(-1.5x8°)<br>+0.5(-1.5x48°)	Slight pigmentary changes in macula	Protan defect	Photopic: no response<br>Scotopic: normal	No	Yes (during first year of life)	Yes	BCM	Multigene (n=4)<br>LW(prox.): 'LIAVA'<br>MW(distal): 'LIAVA'/ 'MVVVA$^{c.465C}$'
BCM133-20960	10 years	0.6<br>0.5	-5.00(-3.75x25°)<br>-4.00(-3.25x160°)	Peripapillary atrophy related to myopia	Moderate red/green axis in both eyes	Photopic / 30 Hz flicker: reduced by 15%<br>Scotopic: normal	Moderate photophobia	Fine, inconstant nystagmus	No apparent progression (in 3 years follow-up)	BCM	Multigene (n=2)<br>LW(prox.): 'LVAVA'<br>MW(distal): 'LVAIA'
BCM133-20961	32 years	0.6<br>0.4	-20.75<br>-19.00	Atrophic posterior pole related to high myopia, slightly narrowed retinal vessels	ND	Photopic: strongly reduced<br>Scotopic: moderately reduced	Severe photophobia	No	Very moderate progression, maybe due to high myopia	BCM	Multigene (n=2)<br>LW(prox.): 'LVAVA'<br>MW(distal): 'LVAIA'
BCM133-23364	1 year	Strabism	-5<br>-5	ND	ND	Photopic: decreased<br>Scotopic: normal	No	No	No follow up	BCM	Multigene (n=2)<br>LW(prox.): 'LVAVA'<br>MW(distal): 'LVAIA'
ZD547-4544	46 years	0.8<br>0.8	-5.0(-3.75x15°)<br>-6.0(-3.75x175°)	Myopic optic nerve	Impaired;<br>D15/Nagel: several confusions along protan axis	Photopic / 30 Hz flicker strongly reduced and delayed<br>Scotopic: normal	Yes	No	No	XLCD	Multigene (n=2)<br>LW(prox.): 'LVAVA'<br>MW(distal): 'MVAVA'
BCM160-23130	6 years	0.3<br>0.5	-3.75(-5.50x8°)<br>-5.0(-5.0x166°)	Papilla with mild *conus myopicus*, thin retina, no true fovea reflex	Impaired, red/green defect	Photopic / 30 Hz flicker reduced and delayed<br>Scotopic: essentially normal	Subjective mild light sensitive	No	Progressive myopia	BCM	Multigene (n=2)<br>LW(prox.): 'LIAVA'<br>MW(distal): 'MVVVA$^{c.465G}$'

Supplementary Table S1 (continued) - Compilation of patients' demographics, clinical data and diagnosis, and genotypes.

Subject code	Age at (last) clinical examination	VA: OD OS	Refraction: OD OS	Fundus	Color vision	ERG	Photophobia	Nystagmus	Progression	Clinical diagnosis	Exon 3 Genotype(s)
**Multigene LW/MW Gene Array (continued)**											
BCM51-12359	12 years	0.2<br>0.2	-14.5(-2.00x50°)<br>-14.5(-1.50x130°)	Myopic discs with peripapillary crescent, stretched and attenuated retinal vessels, thinned RPE in the posterior pole and macula, and fine RPE mottling with granularity and early lacunar or coin-shaped lesions in the mid-periphery, OU.	Protan defect consistent with incomplete achromatopsia	Photopic: DA cone responses to red stimulus, photopic single flash and 30Hz flicker responses strongly subnormal and delayed. Spectral LA cone ERG supports normal distribution of cones<br>Scotopic: mildly subnormal b-waves amplitudes	ND	Small amplitude nystagmus	ND	XLCD	Multigene (n=3)<br>LW(prox.): 'LIAVA'<br>MW(distal): 'MVVVA$^{c.465G}$'
ZD314-18057	10 years	0.2<br>0.2	-6.5(+1.25x100°)<br>-6.0(+2.25x75°)	Pale disk, no foveal reflex, no pigmentary abnormalities	Impaired color vision, no specific axis	Photopic / 30Hz flicker strongly reduced and delayed<br>Scotopic: normal	Yes	No	No	XLCD	Multigene (n=2)<br>LW(prox.) 'LIAVA'<br>MW(distal): 'MVVVA$^{c.465C}$'
BCM98-19713	23 years	0.3<br>0.4	-2.75(-3,5x5°)<br>-0.25(-2.75x180°)	Normal	D-15/saturated: normal;<br>D-15/desaturated: color confusion w/o specific color confusion axis	Photopic / 30Hz flicker response reduced<br>Scotopic: normal	No	No	No	XLCD	Multigene (n=2)<br>LW(prox.): 'LIAIA'<br>MW(distal): 'MVAIA'
BCM142-21958	41 years	0.25<br>0.20	ND	Temporal optic atrophy, no bone spicules or pigment deposits	Impaired;<br>F-M: several confusions along deutan axis	Photopic / 30Hz Flicker strongly reduced and delayed<br>Scotopic: normal	Yes	ND	Yes	BCM	Multigene (n=3)<br>LW(prox.): 'LVAISS'<br>MW(distal): 'MVAIA'/'LVAIA'

Supplementary Table S1 (continued) - Compilation of patients' demographics, clinical data and diagnosis, and genotypes.

Subject code	Age at (last) clinical examination	VA: OD OS	Refraction: OD OS	Fundus	Color vision	ERG	Photophobia	Nystagmus	Progression	Clinical diagnosis	Exon 3 Genotype(s)
**BCM72 family**											
BCM72-16874 (BCM72-I:1)	71 years	0.1<br>0.1	+1.75(-2.5x91°)<br>+0.75(–0.5x95°)	Macular dystrophy	D-15/desat.: Deuteranomaly	normal	No	No	Yes	Deutan/ Macular dystrophy	Multigene (n=4) LW(prox.): 'LIAVS' MW(distal):: 'LIAVA'/ 'MVVVA$^{c.465C}$'
BCM72-17075 (BCM72-III:1)	12 years	0.1<br>0.1	+0.5(-1.5x8°)<br>+0.5(-1.5x48°)	Slight pigmentary changes in macula	D-15/desat.: Protan defect	Photopic: no response<br>Scotopic: normal	No	Yes (during first year of life)	Yes	BCM	Multigene (n=4) LW(prox.): 'LIAVA' MW(distal): 'LIAVA'/ 'MVVVA$^{c.465C}$'
BCM72-16876 (BCM72-II:1)	31 years	0.7 amblyop<br>1.0	-0.25<br>-0.75(-1.0x157°)	No data	D-15/desat.: Normal	Fullfield ERG: normal; multifocal ERG: slightly reduced	No	No	No	normal sighted	female carrier

D-15 – Farnsworth Panel D-15, DA – dark-adapted, F-M – Farnsworth Munsell, LA – light-adapted, ND – no data, OD – *oculus dexter* (right eye), OS – *oculus sinister* (left eye), OU – *oculus uterque* (both eyes), prox. – proximal, RPE – retinal pigment epithelium, VA – Visual acuity, prox.: proximal.

Supplementary Table S2- Physical location and genetic map distances of the microsatellite markers used for segregation analysis.

Microsatellite marker	Marshfield genetic map distance (cM)	Physical distance (bp) according to GRCh37/hg19 assembly
DXS8011	98.2	-
DXS8103	100.73	-
DXS1356	-	chrX:152,698,178-152,698,407
DXS8087	102.35	-
L441TA	-	chrX:153,747,967-153,748,233
L441CA	-	chrX:153,754,802-153,755,044
AF277A	-	chrX:153,778,324-153,778,552
AF277B	-	chrX:153,828,907-153829042
DXS1073	102.35	--

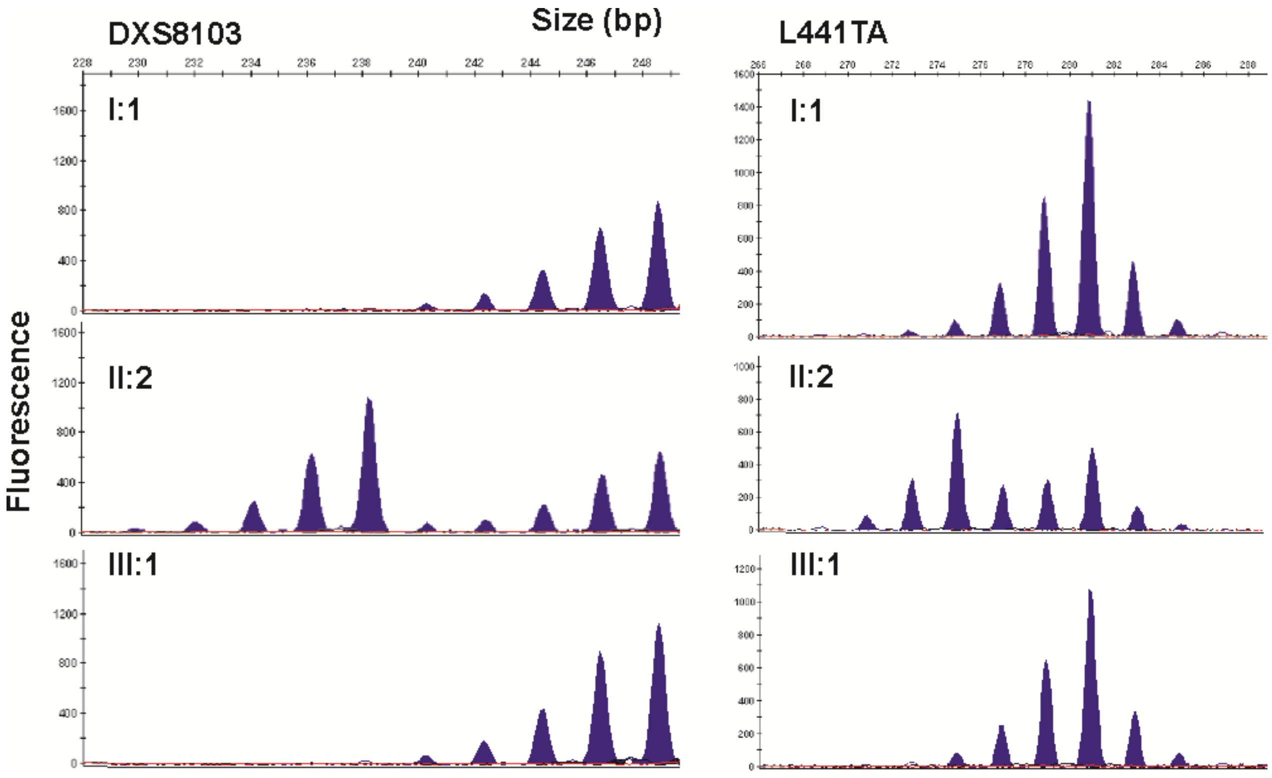

Supplementary Figure S1: Segregation of two informative microsatellite markers demonstrating transmission of the grandparental X-chromosome (BCM72-I:1) to the grandson (BCM72-III:1) in family BCM72. DXS8103 maps centromeric to the *LW/MW* gene cluster, whereas L441TA is the closest marker telomeric to the *LW/MW* gene cluster.

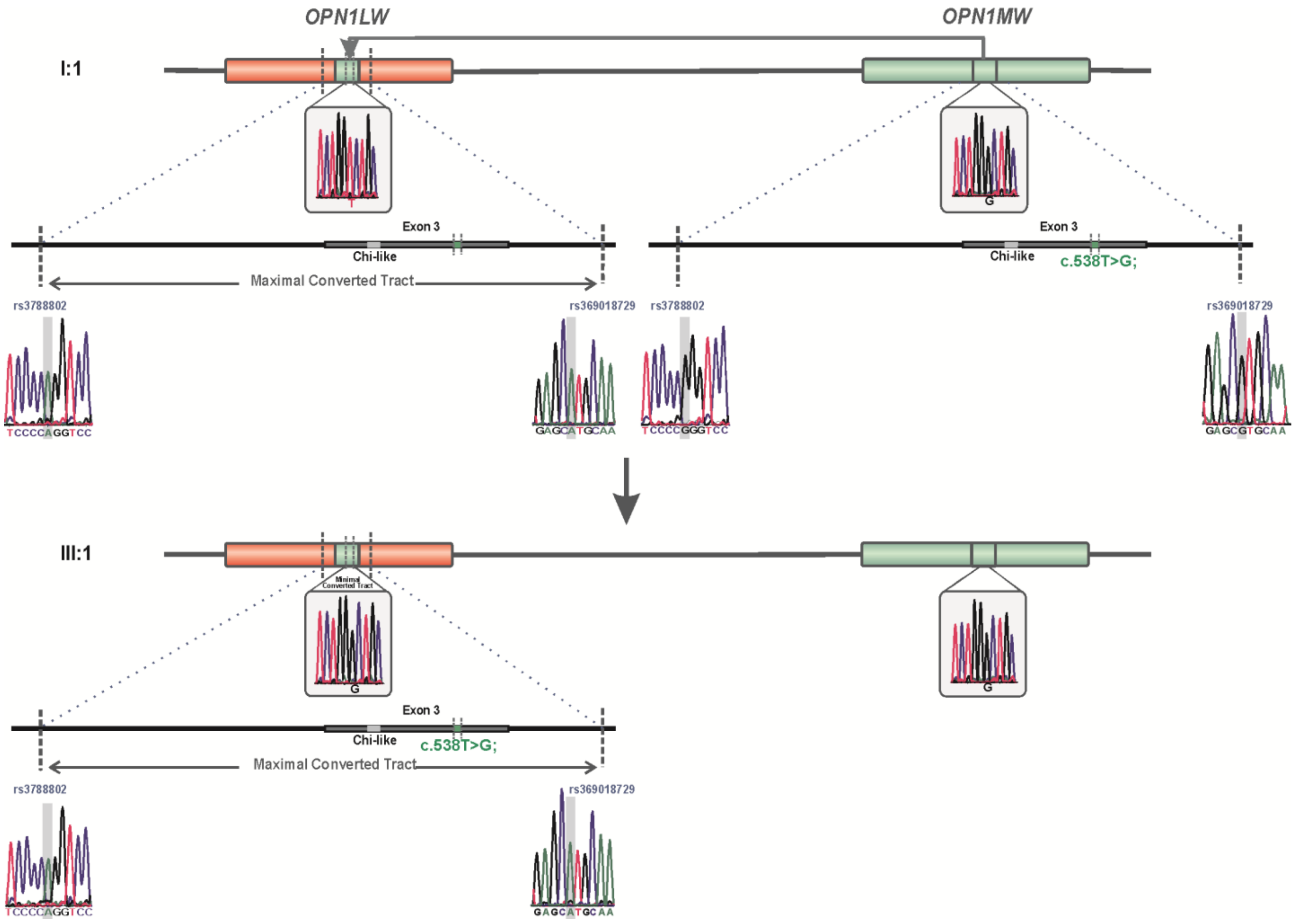

Supplementary Figure S2: Mapping of the outermost borders of the gene conversion event in family BCM72. The maximal converted tract in family BCM72 is delimited by SNPs rs3788802 (c.409+949G>A) in intron 2 and rs69018729 (c.578+91G>A) in intron 3, for which the 'LIAVA' bearing *MW* gene copy in BCM72-I:1 differs from the *LW* gene copies in subjects BCM72-I:1, BCM72-II:2 and BCM72-III:1. Note that the gene conversion event converts the 'LIAVS' bearing *LW* gene copy in BCM72-I:1 into a 'LIAVA' bearing *LW* copy that is transmitted to the mother and the grandson.

Supplementary Figure S3

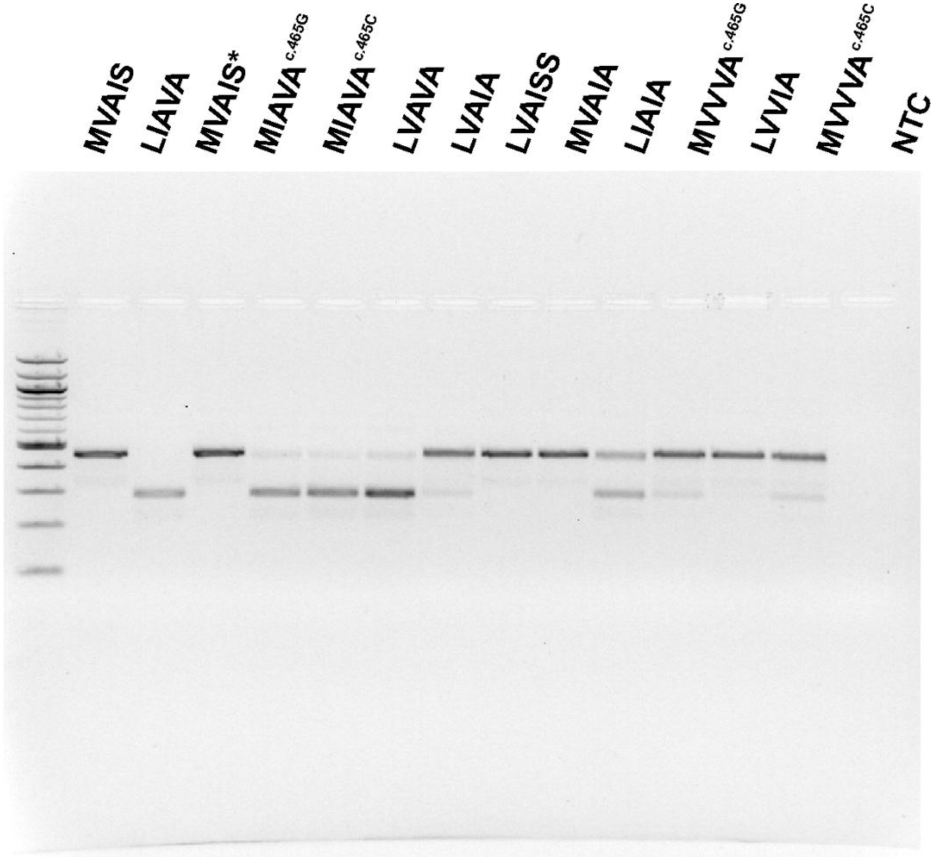

Supplementary Figure S3: Full size gel image of Figure 2a.
Agarose gel electrophoresis of RT-PCR products obtained with RNA from HEK293 cells transfected with minigene constructs bearing various exon 3 haplotypes. The tested haplotype is given above the corresponding gel lane. A 100 bp ladder size standard was loaded in the leftmost lane. Both lanes ‘MVAIS’ and ‘MVAIS*’refer to minigenes carrying the control haplotype. ‘MVAIS*’ has a modified Multiple Cloning Site from the prototype construct ‘MVAIS’ (see Supp. Materials and Methods). NTC: non-template negative control. A scheme on the composition of the RT-PCR products is given on the right.

Supplementary Figure S4

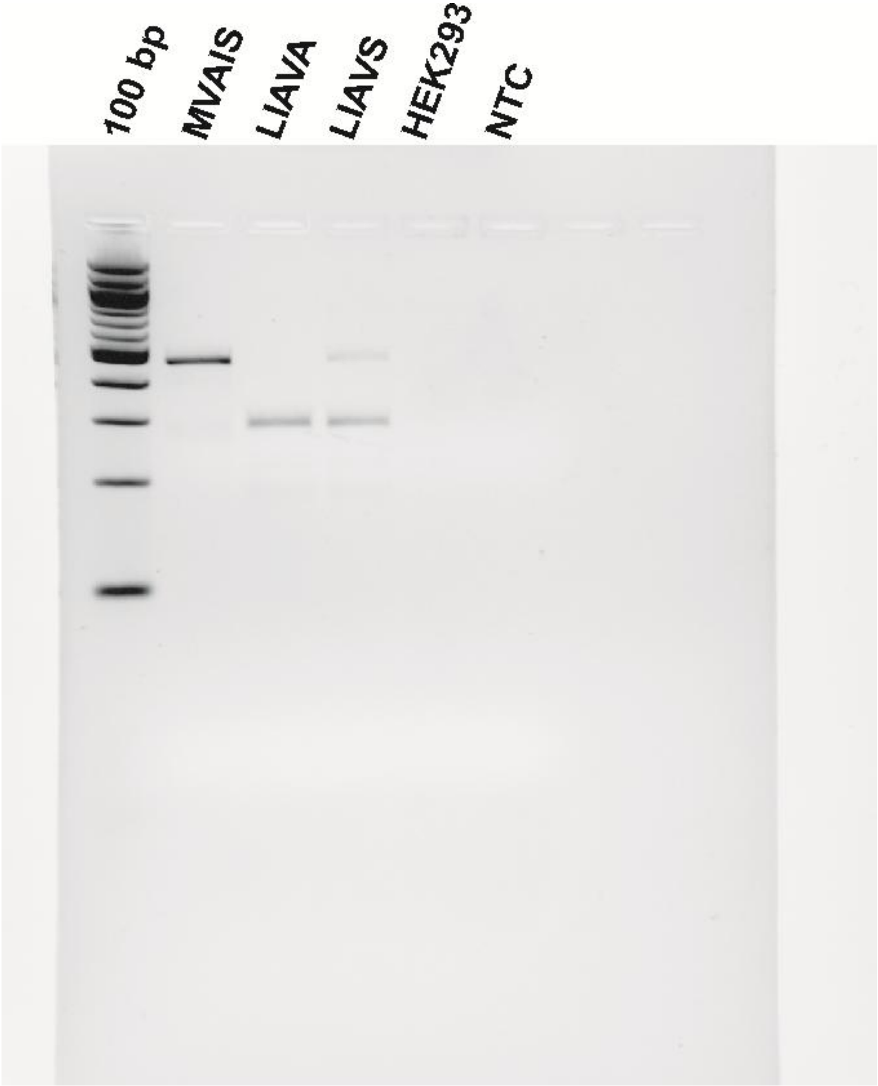

Supplementary Figure S4: Full size gel image of Figure 5a
Direct comparison of RT-PCR products from the minigene splicing assays shows a substantial amount of correctly spliced transcripts (450 bp) for the ‘LIAVS’ exon 3 haplotype, whereas such products are undetectable for the ‘LIAVA’ haplotype. No products were obtained in the RT-PCR with RNA from untransfected HEK293 cells (lane ‘HEK293’) and the no template control PCR (lane ‘NTC’).
